# Rewilding abandoned farmland has greater sustainability benefits than afforestation

**DOI:** 10.1038/s44185-022-00009-9

**Published:** 2023-02-08

**Authors:** Lanhui Wang, Pil Birkefeldt Møller Pedersen, Jens-Christian Svenning

**Affiliations:** 1 Center for Biodiversity Dynamics in a Changing World (BIOCHANGE) and Section for Ecoinformatics & Biodiversity, Department of Biology, Aarhus University, Ny Munkegade 114, 8000 Aarhus C, Denmark; 2 Department of Physical Geography and Ecosystem Science, Lund University, Lund, Sweden; 3 Center for Sustainable Landscapes under Global Change (SustainScapes) and Section for Ecoinformatics & Biodiversity, Department of Biology, Aarhus University, Ny Munkegade 114, 8000 Aarhus C, Denmark

**Keywords:** Ecology, Agroecology, Biodiversity, Climate-change ecology, Restoration ecology

## Abstract

Expansive farmlands in Europe and elsewhere are either already abandoned or projected to become abandoned. Afforestation on these abandoned farmlands is highly popular, but it only addresses the climate crisis, not the biodiversity emergency. An alternative to afforestation is rewilding, which would contribute to combating both the biodiversity and climate crises while also facilitating socio-ecological sustainability by increasing ecosystem resilience.

Low-productivity croplands and pasturelands are being widely abandoned at a global scale, especially in mountainous and remote areas. In the European Union (EU) alone, approximately 11% (more than 20 million ha) of agricultural land is projected to have a high probability of being abandoned during 2015–2030, of which more than 70% is arable land and 20% is pastureland^1^. Drivers of agricultural land abandonment are complex but are generally linked to insufficient incomes from increasing agricultural market competition, urbanisation, population ageing, and soil degradation^2^. Moreover, a proposed dietary shift to more plant-based foods to reduce greenhouse gas emissions could further accelerate the abandonment of farmlands as pastures and arable land will become unnecessary for livestock fodder production^3^. Meanwhile, landowners and managers are increasingly resorting to land intensification through mechanisation or expanding farmland areas where possible^4^. However, these options are not economically or environmentally feasible on marginal and remote land^4^, nor do they represent solutions aligned with international ambitions to combat biodiversity decline and climate change^5^.

A widely used substitute to abandoned farmlands is afforestation, often via planting stands of single or small sets of non-native and native tree species of forestry interest, as opposed to the often slower natural regeneration of native woodland. More than 10^6 ^ha of former cropland have been converted into tree plantations with subsidies from the EU Common Agricultural Policy (Directorate-General for Agriculture and Rural Development 2012). Most of them are Eucalyptus or pine plantations concentrated in the Mediterranean. Management overheads are relatively low compared to long-term revenue from timber sales, and planting trees for carbon sequestration to mitigate anthropogenic climate change has become popular globally^6^. However, such afforestation is ineffective in solving the global biodiversity crisis^6^. For example, subsidised afforestation on abandoned vineyards in Spain has been heavily criticised due to adverse impacts on local biodiversity and ecosystem services^7^. Overall, afforestation only mitigates one part of the dual crises and can often only bring questionable climate mitigation and social benefits, e.g., due to increased fire risk^8,9^ and limited additional soil carbon sequestration^10^, among other things. Such natural and financial or investment risks related to afforestation are expected to increase with climate change but are rarely or unconsidered in current policies and afforestation projects^11^.

## Rewilding is a more sustainable solution

Instead, we argue that rewilding, defined as restoration to promote complex self-sustaining ecosystems by restoring natural ecological factors and processes while reducing human control^9,12^, provides an ecologically, climatically, and socially more sustainable alternative to afforestation on abandoned farmlands in Europe and elsewhere globally. Practical rewilding approaches tailored to different local environments (e.g., the existence of or proximity to relatively intact natural ecosystems) can range from active trophic rewilding (restoring top-down trophic interactions through introductions of lost animal species) to passive rewilding (no or little human intervention from the start)^9^, i.e., especially recommendable where spontaneous recolonisation of ecologically important species is likely. In practice, rewilding applies not only to newly abandoned farmland but also to existing conservation areas with impaired ecological functionality, established forest plantations^13^, or even agricultural areas^7^. Private rewilding initiatives on abandoned farmlands will often be linked to other businesses, such as meat production and eco-tourism, or subsidised through agri-environmental climate schemes. The latter is currently challenged by the EU Common Agricultural Policy (GrazeLIFE report 2021), but after Brexit, the UK is transforming its subsidy schemes, making them more compliant with rewilding approaches (DEFRA 2020). Future business revenue could be coupled with the growing carbon offset market. The possibilities are context-dependent on the local and regional histories of land use, policies, and ownership. Here, we emphasise that rewilding abandoned farmlands should be taken as an opportunity for more holistic ecosystem restoration benefiting the long-term resilience and sustainability of biodiversity, climate, and human livelihoods.

## Biodiversity capacity is higher in rewilded ecosystems

Many abandoned lands associated with low-intensity farming or semi-natural grasslands host high biodiversity in Europe and elsewhere globally^7^. However, following the afforestation of these areas, much biodiversity and ecosystem services (e.g., pollination) are at risk of being lost. For example, cropland afforestation based on coniferous species in southern Europe can cause severe damage to open habitat bird species from increased risk of predation^14^. Furthermore, although plantation forests are habitable for some species, they offer much lower-quality habitats than existing semi-natural grasslands and naturally regenerated forests due to their simplified structure, which is often exacerbated by periodic clear-cutting or the risks of massive fires^11^. In contrast, the biodiversity capacity is higher in rewilded than in afforested landscapes resulting from dynamic interacting processes of trophic complexity and natural disturbances, leading to greater habitat and resource heterogeneity, and stronger dispersal processes^9^. Notably, disturbances to vegetation succession or alternative stable states^15^ created by large herbivores are essential to creating and maintaining heterogeneous vegetation mosaics and associated high levels of biodiversity in many settings^16^. Rewilding on abandoned farmlands will to a varying extent compromise the goal of restoring biodiversity due to business model considerations (e.g., optimal meat production or eco-tourism), but these compromises are minor compared to the potential harm to biodiversity that may be caused by high-productivity afforestation schemes.

## The climate change mitigation potential of afforestation can be less favourable than rewilding

Above-ground carbon sequestration rates of afforested forest plantations using fast-growing tree species can be higher than those of passive rewilding areas, but the trees will eventually be harvested. Although long-lived harvested wood products can be substantial carbon sinks in some countries under certain socio-economic conditions, the carbon stored annually in harvested wood products amounts to less than 1% of global carbon emissions^17^. Moreover, wood harvest and re-planting will require CO_2_-emitting machinery and cause some of the captured carbon in the topsoil to become lost again in run-off. Forestry together with agriculture and other land uses, is responsible for a substantial (23%) part of net annual anthropogenic greenhouse gas emissions^18^. Finally, the importance of afforestation in increasing soil organic carbon may be overestimated^19^.

Furthermore, trees not only influence the climate by sequestering CO_2_ during photosynthesis, but also their dark green crowns absorb solar radiation (warming effect), and they suck up water from soils and evaporate it from their leaves (i.e., evapotranspiration) which creates clouds that lower the absorption of solar radiation (cooling effect). Therefore, planting trees in tropical regions increases the overall albedo, as evapotranspiration will cause clouds. In contrast, in high-latitudinal regions with seasonal snow cover, planting trees or woody encroachment caused by land abandonment will decrease the overall albedo since dark tree crowns absorb more solar radiation than snow-covered ground and contribute to regional and global warming^20^. In fact, climate change mitigation potential through global dryland forestation is found to be limited, although many re-afforestation projects are targeting these often naturally open ecosystems^21^.

Finally, high-latitude and alpine permafrost soils hold substantial amounts of carbon that will be decomposed and emitted into the atmosphere as CO_2_ and methane if thawed, so warming-induced permafrost thawing will start a positive feedback mechanism releasing much more greenhouse gases. There is evidence from high latitudes that the sheer presence and trampling activity of large herbivores can reduce snow depths, decrease snow insulation capacity, and lower soil temperature in permafrost during winter, preventing or slowing permafrost thawing^22^. The prospects of climate change mitigation capacity of rewilding projects in tundra regions, including (re)introductions of large herbivores in abandoned grazing pastures and woody encroached areas, are promising and should be tested and quantified at regional to continental scales.

## Rewilded ecosystems are more resilient to increasing climate pressures

Plantation forests differ from old-growth intact forests, partly due to their uniform, simplified structure and reduced functional resilience^6^. In addition, trees are typically harvested after 10 to 60 years, causing periodic collapse of regenerating biological communities. Moreover, forest plantations and stored above-ground carbon in water-constrained areas are likely to suffer more from enhanced fire regimes induced by climate change and increasing climate extremes, especially droughts and heatwaves^8,11^.

In contrast, rewilded ecosystems are expected to be more resilient to increasing warming-induced climate extremes in maintaining biodiversity and carbon sinks than forest plantations. Heterogeneous microhabitats and dispersal dynamics, created and maintained by natural disturbances and fostered by large animals in rewilded landscapes, are crucial for diverse species to survive and recover rapidly from climate extremes^8^, enhancing the overall adaptive capacity of biodiversity and ecosystem resilience. Rewilded landscapes are also vital for carbon persistence under extreme droughts causing a high incidence of wildfires^8^. As in natural fire-prone savannas and boreal forests, restored assemblages of large herbivores are likely to reduce wildfire risk^23^ through biomass consumption and promoting spatial heterogeneity and discontinuity of vegetation cover, facilitating long-term maintenance of sequestered carbon in above-ground woody biomass. Moreover, the (re)introduction of large herbivores into rewilding landscapes will likely increase total carbon storage and persistence by redistributing carbon from above-ground disturbance-vulnerable vegetation into permanent below-ground soil pools^24^.

## Rewilding is more cost-effective and societally beneficial

Rewilding is expected to be more socio-economically cost-effective because of its non- or low-intervention principles and unique upscaling potential, much needed for restoring large-scale abandoned landscapes^12^. Specifically, rewilding relies on natural biotic processes, such as grazing and browsing by large herbivores, to create habitat heterogeneity and plant dispersal dynamics – these fauna-mediated processes are intrinsically scalable through natural population growth and expansion. In contrast, both the economic costs associated with the initial investment and the long-term stewardship can be high for afforestation programs and the operation of forest plantations.

With low levels of public investment or philanthropic donations, rewilded landscapes can provide and improve various ecosystem services, particularly regulating (e.g., water purification and buffering) and cultural services (e.g., recreation and nature therapy), which are long-term services that are often prioritised subordinately to short-term economic outcomes by individual landowners. Furthermore, the otherwise diminished but important cultural heritages associated with low-intensity traditional farming can be maintained in a potentially transformed manner, along with rewilding through a nature-based sustainable economy. For example, rewilded landscapes can create new businesses and job opportunities (e.g., eco-tourism and sustainable game hunting) and attract surrounding urban residents to re-engage with nature^12^. For privately owned land, a profitable (logically as profitable as those for afforestation) and sustainable business model is vital to mobilise the willingness of farmers to participate in rewilding schemes and to ensure their long-term commitment, on which the success of these strategies depends^25^. Therefore, both locals and people far away can benefit from the positive effects of the recovery of wilder nature and improve the overall quality of life. Nevertheless, challenges such as land tenure, long-term financial schemes, and potential human-wildlife conflicts from rewilding should be well considered and handled in a socio-ecological context – transparently, fairly, and through the empowerment of local people.

## Conclusions and looking forward

In summary, afforestation in unsuitable places, such as naturally open or carbon-rich soils (e.g., peatlands), can be devastating to biodiversity^16^ and work against its climate regulation intentions, reinforcing warming rather than mitigating climate change^20^. In contrast, recovering natural ecosystems through rewilding should eventually maximise their overall carbon sequestration and storage capacity through both above- and below-ground woody biomass and soils^26^. Moreover, rewilded areas are of much greater value to biodiversity and more resilient to increasing climate extremes than forest plantations. Finally, the social benefits of rewilding have the potential to outperform afforestation due to its cost-effectiveness and the multitude of ecosystem services and livelihood opportunities.

We thus argue that rewilding abandoned farmlands should be promoted and prioritised as one of the most sustainable long-term solutions to the dual crises of biodiversity loss and climate change. However, given the complexities of land use change and social, cultural and economic histories in the land being rewilded, the success of different rewilding practices will always be context-dependent and must be adaptable across space and time. Here, the newly developed general guiding principles for rewilding by the International Union for Conservation of Nature (IUCN) can serve as a good reference in practice^27^. In particular, rewilding projects should include continuous ground and space-based monitoring of habitat heterogeneity, species diversity dynamics, and nature’s contributions to people at multiple spatio-temporal scales to enable timely and effective resilience-based adaptive management interventions when needed. Furthermore, in the context of the United Nations (UN) Sustainable Development Goals and the Decade on Ecosystem Restoration, the implementation of rewilding in global and national policies should be facilitated and coordinated through the three UN environmental conventions on biodiversity, climate change, and land degradation. Specifically, rewilding initiatives should be supported by subsidy schemes from the EU Common Agricultural Policy or other new financial institutions or financed through investments associated with the carbon offset market.

Finally, to further promote rewilding as a socio-ecological landscape recovery strategy, we recommend collaboration between scientists, policymakers, decision-makers, and local communities to co-design sustainable ecosystem stewardships providing economic support to farmers to transit from intensive farmland management to rewilding. This will be an essential step to ensure a successful transformative change of agriculture and rural communities toward the long-term socio-ecological sustainability of anthropogenic landscapes and planet Earth.
